# Characterizing heterogeneity and subphenotyping acute respiratory distress syndrome with computed tomography

**DOI:** 10.1186/s40635-026-00880-x

**Published:** 2026-03-26

**Authors:** Roberta Garberi, Matthieu Jabaudon, Sam Bayat, Sarah E. Gerard, Aurora Magliocca, Mariangela Pellegrini, Alberto Bravin, Lorraine B. Ware, John J. Marini, Yi Xin, John G. Laffey, Maurizio Cereda, Emanuele Rezoagli

**Affiliations:** 1 School of Medicine and Surgery, University of Milano-Bicocca, Monza, Italy; 2 Department of Perioperative Medicine, CHU Clermont-Ferrand and iGReD, CNRS, INSERM, Université Clermont Auvergne, Clermont-Ferrand, France; 3 Department of Pulmonology & Physiology, Grenoble University Hospital, Grenoble, France; 4 INSERM UA7, STROBE Laboratory, University of Grenoble Alpes, Grenoble, France; 5 Roy J. Carver Department of Biomedical Engineering, University of Iowa, Iowa City, USA; 6 Department of Pathophysiology and Transplantation, University of Milan, Milan, Italy; 7 Intensive Care Unit, Department of Anaesthesia, Operation and Intensive Care, Uppsala University Hospital, Uppsala, Sweden; 8 Anesthesiology and Intensive Care Medicine, Department of Surgical Sciences, Uppsala University, Uppsala, Sweden; 9 Dipartimento Di Fisica, Università Degli Studi Di Milano-Bicocca, Milan, Italy; 10 Dipartimento Di Fisica, Università Della Calabria, Arcavacata di Rende, Italy; 11 Istituto Nazionale Di Fisica Nucleare, Sezione Di Milano-Bicocca, Milan, Italy; 12 CNR-Nanotec, Sezione di Rende, Italy; 13 Department of Medicine, Vanderbilt University Medical Center, Nashville, TN, USA; 14 Department of Pathology, Microbiology and Immunology, Vanderbilt University Medical Center, Nashville, TN USA; 15 Division of Pulmonary and Critical Care Medicine, Regions Hospital, University of Minnesota, Minneapolis, MN USA; 16 Anesthesia Center for Critical Care Research, Department of Anesthesiology, Critical Care and Pain Medicine, Mass General Brigham and Harvard Medical School, Boston, MA USA; 17 Regenerative Medicine Institute at CÚRAM Centre for Research in Medical Devices, Biomedical Sciences Building, and Discipline of Anaesthesia, School of Medicine, National University of Ireland Galway, Galway, Ireland; 18 Department of Anaesthesia and Intensive Care Medicine, Clinical Sciences Institute, Galway University Hospitals, and School of Medicine, National University of Ireland, Galway, Ireland; 19 Department of Emergency and Intensive Care, Fondazione IRCCS San Gerardo Dei Tintori, Monza, Italy

**Keywords:** Acute respiratory distress syndrome (ARDS), Quantitative computed tomography (qCT), CT-based subphenotyping, Ventilation–perfusion mismatch, Artificial Intelligence (AI)

## Abstract

Acute respiratory distress syndrome (ARDS) is a heterogeneous clinical syndrome rather than a single disease. Patients who meet the same diagnostic criteria may differ in lung morphology, mechanical properties, biological injury, and clinical course. Current classifications rely largely on the severity of hypoxemia and do not capture this variability, limiting prognostic stratification and individualized treatment. This heterogeneity has clinical consequences. Supportive interventions such as positive end-expiratory pressure (PEEP), prone positioning, and recruitment maneuvers are broadly applied, yet their effects vary substantially among patients. Increasing evidence indicates that these differences are partly explained by variation in lung structure, regional aeration, recruitability, and perfusion. Recent international guidelines have identified phenotyping as a priority in ARDS and have highlighted lung morphology as a relevant source of prognostic enrichment and treatment effect heterogeneity. Computed tomography (CT) provides regional, three-dimensional information on lung injury that is not accessible through bedside physiological measurements. It allows evaluation of aeration loss, lung density, lung weight, and perfusion abnormalities. CT has been used to describe key aspects of lung injury in ARDS and to identify imaging patterns associated with lung mechanics, gas exchange, and response to ventilatory settings. Quantitative and dual-energy CT, together with computational methods, allow a more detailed description of these patterns. This review examines the role of CT in characterizing heterogeneity in ARDS, summarizes qualitative, semi-quantitative, and quantitative approaches, and discusses their clinical relevance and limitations, as well as future directions.

## Take-home message

**Table:** 

Acute respiratory distress syndrome (ARDS) is a heterogeneous clinical syndrome in which lung morphology, aeration, and perfusion vary across patients and are not fully reflected by conventional severity criteria. CT-driven phenotyping, from visual assessment to quantitative and dual-energy CT, provides regional information on lung structure and injury that complements bedside physiological measurements and supports a more refined description of ARDS heterogeneity.	

## Introduction

Acute respiratory distress syndrome (ARDS) is a severe form of acute respiratory failure (ARF), first described in 1967 [1]. It arises from heterogeneous etiologies and encompasses a broad spectrum of pathophysiological mechanisms [2]. Despite decades of research and progress in supportive care, the mortality rate remains high [3].

The Berlin Definition stratifies ARDS severity based on oxygenation, referring to the partial pressure of arterial oxygen-to-fraction of inspired oxygen (PaO_2_:F_I_O_2_) ratio, all on a minimum positive end-expiratory pressure (PEEP) of 5 cmH_2_O [4]. However, the prognostic performance of oxygenation remains limited, and a recent analysis of the LUNG SAFE database confirmed its poor outcome discrimination [5]. Indeed, the Berlin definition does not account for the underlying biological processes, mechanical properties or morphological patterns of injury, which are now recognized to be heterogeneous within this syndrome [6]. This heterogeneity has major clinical implications. Supportive interventions such as low tidal volume ventilation [7], prone positioning [8] and high PEEP strategies [9, 10] show benefits in ARDS cohorts [11], but not all patients seem to respond equally [7, 12, 13]. For example, higher PEEP improves oxygenation and outcomes in patients with recruitable lungs but may cause overdistension and harm in those with low recruitability [14, 15].

Recent guidelines highlighted the challenge of recommending uniform supportive interventions for the entire ARDS population [16, 17]. For the first time in an intensive care-related guideline, the 2023 ESICM guidelines explicitly include phenotyping as a key research and clinical priority domain, highlighting lung morphology as one of the sources of prognostic/predictive enrichment and treatment effect heterogeneity within ARDS populations [17].

Over the past two decades, advances in lung imaging by computed tomography (CT), such as quantitative CT (qCT) and dual-energy CT (DECT), together with bedside tools such as electrical impedance tomography (EIT) and lung ultrasound (LUS), have improved the characterization and quantification of aeration, consolidation, edema, and perfusion. These techniques have been used to describe imaging-based patterns and to explore “imaging subphenotypes” [18, 19].

This review aims to synthesize current evidence on CT-driven subphenotyping in ARDS. We integrate qualitative, semi-quantitative, and qCT analysis, including dynamic CT, DECT, and photon counting CT (PCCT) and describe their current applications. We discuss how distinct morphologic and perfusion patterns relate to physiological impairments, lung recruitability, and differential response to ventilatory strategies, and we highlight translational implications, methodological limitations, and future research priorities. Given the increasing use of CT-based metrics in ARDS studies, this structured overview may help contextualize current findings, highlight methodological heterogeneity, and identify areas where evidence remains limited.

## Quantitative CT: principles and rationale

When ARDS was first defined, diagnosis relied on the presence of new-onset bilateral opacities on chest radiograph (chest X-ray). Subsequent analyses showed that mortality increased stepwise with the number of radiographic lung quadrants involved, and that unilateral infiltrates carried mortality comparable to ARDS with two infiltrated quadrants in patients undergoing controlled mechanical ventilation [20]. This finding was recently replicated in patients with COVID-19 acute respiratory failure during spontaneous breathing [21].

These observations underscore that the extent and distribution of opacities matter. Consistently, semi-quantitative chest radiograph scores that incorporate both the extent and density of alveolar opacities, such as the Radiographic Assessment of Lung Edema (RALE) score, have been shown to correlate with oxygenation and to be independently associated with mortality in ARDS cohorts. However, the chest X-ray is a two-dimensional projection of a three-dimensional organ and, therefore, it does not capture the lung’s complex spatial heterogeneity. As a result, it often underestimates the true extension of consolidation, edema, and loss of aeration, particularly in dependent regions [22].

Early CT studies of supine ARDS patients revealed a marked ventrodorsal density gradient of aeration: the most dependent regions were typically consolidated, or non-aerated, intermediate zones were poorly aerated, and non-dependent zones were relatively well aerated or hyperinflated [23]. This vertical gradient was shown to be much more pronounced than in healthy lungs, as demonstrated by qCT analysis of regional gas-to-tissue ratios [24]. A major physiological explanation for this phenomenon lies in the “sponge lung” behavior of edematous parenchyma: the lung becomes heavier because of the accumulation of extravascular water, and the weight of this tissue generates superimposed pressure which increases with depth below the sternum. Where this hydrostatic load exceeds airway pressure, dependent alveoli are predisposed to collapse [25, 26].

Prone positioning reverses the gravitational distribution of aeration: dorsal regions, which in the supine position are dependent and consolidated, become non-dependent and reexpand, while ventral areas tend to increase in density [27]. These shifts were shown to occur without changes in PEEP, underscoring that much of the opacity pattern in ARDS reflects gravity- and weight-dependent compression rather than fixed, irreversible pathology or anatomic variation [28].

In ARDS, CT scan analysis also demonstrated that the amount of well-aerated tissue is markedly reduced compared with healthy lungs [29]. This led to the formulation of the “baby lung” concept, which implies that inflatable lung units are not intrinsically stiff: the specific compliance of the aerated lung remains almost normal, but the monitored respiratory system compliance is low because tidal volume is applied to a small fraction of lung [30, 31]. The consequence is that regional stress and strain are disproportionately high, predisposing to ventilator-induced lung injury (VILI). Importantly, the “baby lung” is a functional rather than an anatomical entity, as its size and distribution vary dynamically with positioning and ventilatory settings [31, 32]. To avoid overdistension, these observations support tailoring protective ventilation to the size of the “baby lung” rather than adjusting it to predicted body weight [29, 33, 34]. Likewise, CT-defined patterns are not static. Lung morphology can change over time and in response to ventilatory adjustments or disease progression, and imaging phenotypes should therefore be interpreted within the overall clinical trajectory of the patient. Although prospective data are limited, serial CT imaging may help better characterize these changes and support ventilation decisions as the patient’s condition evolves.

**Fig. 1:**
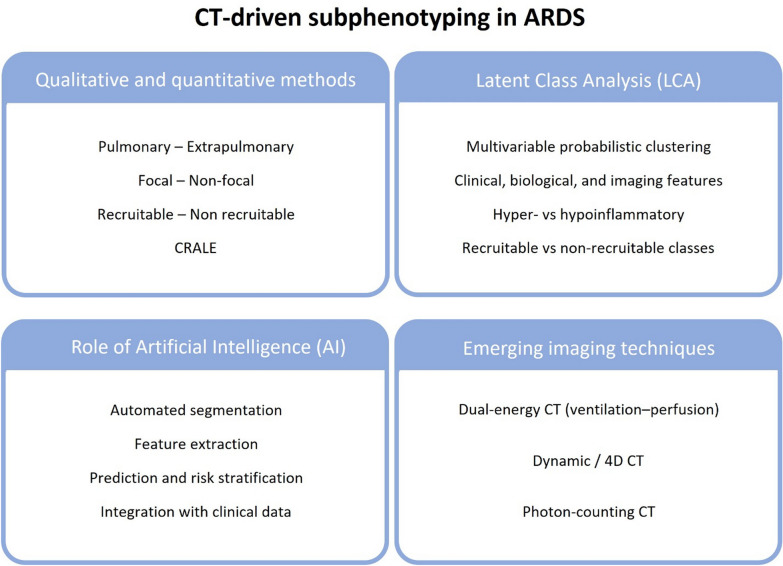
General overview of the key topics

Collectively, these observations established qCT as a framework to describe ARDS heterogeneity in spatial and functional terms (Fig. 1), enabling the identification of morphologic and functional subphenotypes with potential implications for ventilatory management, as outlined below.

### Pulmonary and extrapulmonary ARDS

Early physiological and CT-based descriptions suggested that pulmonary ARDS, often due to pneumonia or aspiration, more frequently shows asymmetric, focal consolidation, whereas extrapulmonary ARDS, more commonly linked to systemic insults such as pancreatitis or extrapulmonary sepsis, tends to show a more diffuse and symmetric ground-glass pattern [35]. However, these associations are not absolute, and considerable overlap between etiologic categories and CT patterns has been reported.

These morphological distinctions have physiological implications: focal consolidation is typically associated with lower recruitability and tends to respond poorly to high PEEP, whereas more diffuse involvement often shows greater recruitability and may benefit from recruitment maneuvers [15, 36].

A comparative CT study of 41 ARDS patients reported that “typical” CT patterns, defined as dependent parenchymal opacification merging with non-dependent ground-glass opacities, were seen more frequently in extrapulmonary than in pulmonary ARDS, while “atypical” patterns characterized by more extensive non-dependent opacities and parenchymal cysts were more common in pulmonary ARDS. Multivariable analysis found that the typical pattern was independently associated with an extrapulmonary etiology [37].

To further explore whether the distinction between pulmonary and extrapulmonary ARDS carries prognostic relevance, a single-center Japanese cohort study of 200 patients found no significant differences between the two groups in 60-day mortality or in the probability of successful liberation from mechanical ventilation. In multivariable analysis, outcome was independently associated with the high-resolution CT (HRCT) fibroproliferation score and to the Disseminated Intravascular Coagulation (DIC) score, a composite index of coagulation abnormalities, whereas etiology was not. [38]. The HRCT fibroproliferation score is a semi-quantitative index that integrates the extent of parenchymal opacities with fibrotic features such as reticulation, traction bronchiectasis, and honeycombing across lung zones [39]. Its association with mortality supports the concept that the intensity and nature of lung injury, rather than the initial site of insult, drive prognosis in ARDS.

### COVID-19 ARDS

Early in the COVID-19 pandemic, clinicians observed an apparent dissociation between profound hypoxemia and relatively preserved respiratory system compliance [40, 41]. On this basis, the L (low elastance, low recruitability, low lung weight) and H (high elastance, high recruitability, high lung weight) subphenotypes were proposed as a practical framework for early management and to explain divergent ventilatory responses. Type L was characterized by near-normal compliance, less non-aerated tissue and subpleural ground-glass on CT, whereas Type H resembled severe ARDS with high lung weight, shunt, and recruitability, and was managed as classical ARDS (higher PEEP, prone positioning, extracorporeal support as needed) [42, 43].

Subsequent matched-cohort quantitative CT studies comparing mechanically ventilated COVID-19 ARDS with “typical” ARDS refined this picture: at matched PaO_2_:FiO_2_, early COVID-19 patients exhibited higher compliance, larger end-expiratory gas volumes and less non-aerated tissue; at matched compliance, they had worse oxygenating efficiency. Notably, venous admixture correlated with the fraction of non-aerated tissue in non-COVID ARDS but did not correlate in COVID-19, indicating that hypoxemia was not explained only by collapse/non-aeration and suggesting a stronger perfusion component [44].

Rather than a strict dichotomy, COVID-19 is an evolving continuum in which temporal phase and patient effort influence progression. In early disease, hypoxemia was not as tightly linked to CT-derived lung weight or non-aerated fractions [45]. A submantellar-to-hilar density gradient, characterized by a central progression of injury, was observed in a cohort of 111 COVID-19 ARDS patients following exposure to spontaneous breathing and high tidal volumes, both recognized contributors to P-SILI [46, 47].

Consistent with this continuum, CT series obtained in the early phase of disease documented ground-glass opacities, typically bilateral and peripheral/subpleural, as the predominant presentation, with progression over the first 1–2 weeks to patterns of “crazy paving” and patchy consolidations before their gradual absorption; in survivors, follow-up often revealed fibrotic-like residuals and heterogeneous recovery trajectories [48–51].

In this context, CT-informed patterns further delineated three pragmatic subphenotypes: (1) focal, possibly overperfused ground-glass opacities (vasocentric hypoxemia with limited response to PEEP); (2) inhomogeneous atelectasis (recruitable and responsive to PEEP and prone position); and (3) patchy ARDS-like involvement (managed as classical ARDS) [45]. Collectively, these data caution against oversimplified classifications favoring individualized strategies [45, 52].

### Focal and non-focal subphenotypes

**Fig. 2:**
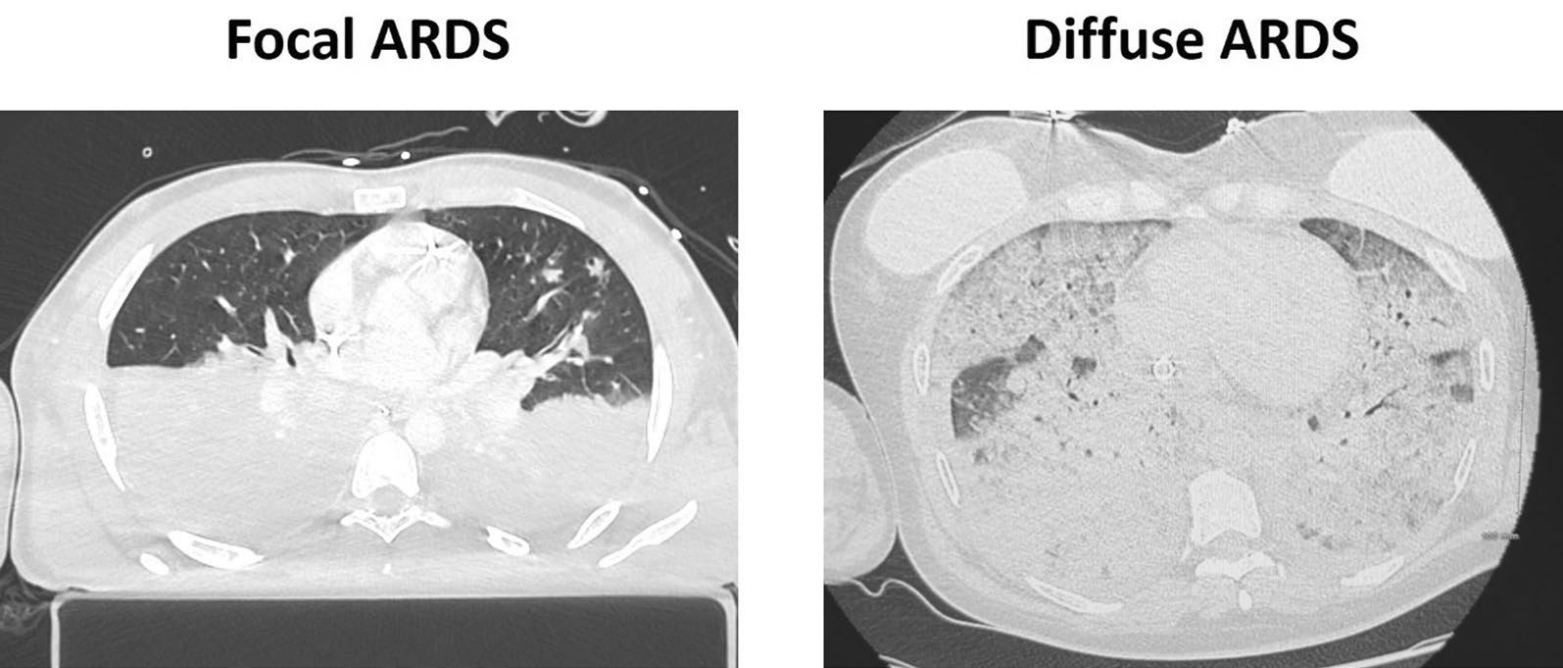
Qualitative subphenotypic differentiation: focal vs. diffuse

Morphologic subphenotyping by thoracic CT has revealed distinct patterns of loss of aeration in ARDS, classically described as focal and non-focal (Fig. 2). In focal ARDS, loss of aeration predominates in the dorsal regions with relative sparing of ventral zones, whereas in non-focal ARDS consolidation and ground-glass opacities are distributed more diffusely across the lung [53, 54]. These radiological patterns relate to recruitability and ventilatory response. Focal ARDS tends to be poorly recruitable and more prone to overdistension when high PEEP or recruitment maneuvers are applied. In such cases, prone positioning and moderate PEEP levels are often considered, with attention to overinflation. In contrast, non-focal ARDS is frequently associated with a greater amount of recruitable lung tissue which can translate into a more evident response when PEEP is increased [15, 53].

The importance of this distinction was tested in the LIVE trial, which attempted to adapt ventilation strategies to focal versus nonfocal morphologies, assessed with either CT or chest X-ray [14]. The trial was limited by a high rate of misclassification and failed to demonstrate a survival benefit. However, it suggested that when morphological classification was correct, patients appeared to benefit from a subphenotype-adapted ventilation strategy, whereas misclassified patients had worse outcomes [14]. Overall, the LIVE trial underscored the need for accurate, reproducible classification.

**Fig. 3:**
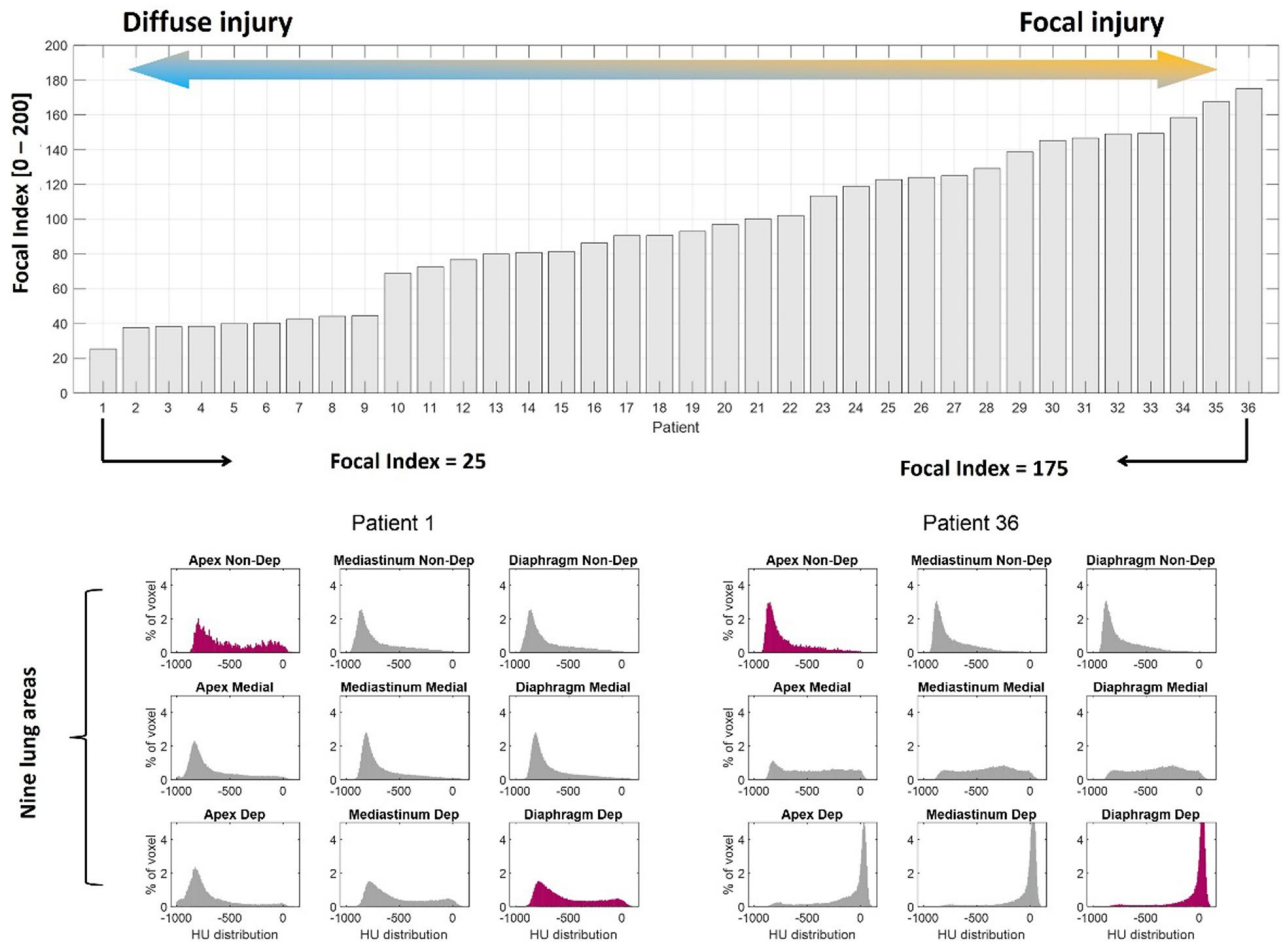
The Focal index was calculated as the size of the remaining non-overlapping AUC (area under the curve) when superimposing the HU histograms of the apical non-dependent and diaphragmatic dependent areas. A Focal index of 0 (complete overlap, indicating the absence of a gradient between the more apical non-dependent and the more basal dependent areas) corresponds to a more diffuse ARDS; a Focal Index of 200 corresponds to focal ARDS. Above: the calculated Focal index from the 36 COVID-19 ARDS patients included in the study, showing a continuous spectrum of values. Below: two extreme examples of regional HU distribution profiles when the lung is divided into nine different areas. Left: in a more diffuse injury, the HU distribution profile is similar among areas; Right: in a more focal injury, the HU distribution profile is very different among areas, with a peak of hyperinflation corresponding to the most apical and non-dependent lung regions and a peak of non-aeration corresponding to the most peridiaphragmatic and dependent lung regions

To reduce subjectivity, quantitative metrics such as the focal index have been proposed [55]. In a recent single-center retrospective pilot study involving COVID-19 ARDS patients, the focal index was introduced as a continuous, numerical measure of how focal or diffuse the lung injury is, as opposed to a subjective dichotomy of focal versus non-focal. The method relies on CT-derived Hounsfield Unit (HU) histograms; density distributions are computed separately for an apical ventral region and for a diaphragm-adjacent dorsal region, and the index corresponds to the non-overlapping area between the two curves. By design, values near 0 reflect almost complete overlap of the two HU distributions and therefore a diffuse pattern; values approaching 200 reflect minimal overlap and therefore indicate a highly focal pattern (Fig. 3). In this pilot cohort, the focal index varied widely across patients, correlated positively with the proportion of dorsal diaphragmatic non-aerated lung and inversely with total gas volume. It did not change with differences in ventilator settings, indicating that it primarily reflects intrinsic injury patterns rather than the results of applied ventilation [55].

Although defined radiographically, the focal versus non-focal classification is supported by biological data. The soluble receptor for advanced glycation end-products (sRAGE) is a marker released into the plasma when the alveolar epithelium, specifically type I cells, is injured. Experimental and clinical data showed sRAGE expression on type I cells and higher circulating sRAGE levels in acute lung injury. In contrast, sRAGE levels were not increased in severe sepsis without lung involvement [56]. In a prospective multicenter ARDS cohort, patients with a non-focal CT pattern had higher circulating plasma sRAGE than those with a focal pattern, and sRAGE reliably separated the two groups. The non-focal subphenotype was also associated with higher 28- and 90-day mortality. Together, these findings indicate that the diffuse CT pattern is typically accompanied by greater lung epithelial injury and a higher risk profile [57].

### Recruitable and non-recruitable subphenotypes

In clinical practice, recruitment metrics aim to quantify how easily non-aerated lung can be reopened by increasing applied pressure and in doing so facilitate rational choices for PEEP, recruitment maneuvers, and positioning. qCT first established that the fraction of potentially recruitable lung varies widely across ARDS patients and that inter-individual differences in recruitability strongly condition the physiological response to higher PEEP.

In a study using whole-lung CT at multiple pressure levels, with recruitability quantified as the reduction in non-aerated lung mass between 5 and 45 cmH_2_O, the amount of lung tissue that became aerated with increasing airway pressure ranged from negligible to extensive and was associated with changes in oxygenation, expressed as PaO_2_:FiO_2_, in response to higher PEEP [15].

Methodologically, CT-based recruitability has been determined in several ways that are conceptually related but numerically distinct (Table 1). These include: (1) variation of non-aerated mass: the reduction in non-aerated lung mass between low and high PEEP [15, 58]; (2) variation of non-aerated plus poorly aerated mass, intended to capture recruitment that transitions through poorly aerated compartments [58, 59]; (3) variation of gas volume: pressure-induced increase in gas within previously non-aerated regions [60], within both poorly and non-aerated regions [61], or as the overall increase in total gas volume [62]. Choices among these definitions and the specific pressure steps mathematically change the absolute recruitability estimate and therefore need to be specified for comparability. In addition, quantitative estimates are sensitive to acquisition parameters such as slice thickness, reconstruction kernel, radiation dose, and segmentation methods. For this reason, standardized imaging and analysis protocols are needed to improve reproducibility and comparability across studies.

**Table 1:** CT–based definitions of lung recruitment used in physiological studies of ARDS.

Study	Methods	CT definition of recruitment	Conditions	Reported metric	Implication for comparability
Gattinoni et al., *N Engl J Med*, 2006 [15]	CT, tissue-based (potentially recruitable lung)	Recruitment defined as the proportion of lung tissue that regains aeration between low and very high airway pressures, estimated from the reduction in non-aerated lung tissue between conditions	Whole-lung CT at 5 and 15 cmH_2_O (end-expiratory), and 45 cmH_2_O (recruitment maneuver)	% potentially recruitable lung	Estimates a maximal recruitable fraction; values are not comparable with studies using smaller PEEP steps
Chiumello et al., *Am J Respir Crit Care Med*, 2016 [58]	CT, tissue-based + respiratory mechanics	CT recruitment quantified as the reduction in non-aerated tissue alone and, alternatively, as the reduction in non-aerated plus poorly aerated tissue between PEEP levels	PEEP 5 vs 15 cmH_2_O	grams or % of lung tissue (depending on normalization)	Demonstrates that different tissue-based definitions produce different recruitment estimates
Del Sorbo et al., *Crit Care*, 2023 [59]	Bedside surrogate (R/I) with CT reference	CT-based lung tissue recruitment used as reference for R/I, calculated according to the Chiumello method as the reduction in the proportion of non-inflated plus poorly inflated tissue relative to total lung weight between PEEP levels	PEEP 5 vs 15 cmH_2_O	% recruitment (normalized to lung weight)	Uses a Δ(NA + PA) tissue definition, not equivalent to ΔNA-only or high-pressure CT protocols
Lu et al., *Crit Care*, 2006 [60]	CT, gas-volume and compartment-based	CT-derived alveolar derecruitment defined as the difference in gas volume present in poorly aerated plus non-aerated lung regions between PEEP levels	PEEP vs ZEEP	mL of derecruited gas/compartment volumes	Shows that changes in total lung gas volume (ΔFRC) do not uniquely reflect alveolar derecruitment, as a large fraction is due to deflation of normally aerated regions
Malbouisson et al., *Am J Respir Crit Care Med*, 2001 [61]	CT, gas-based (compartment-targeted)	Recruitment defined as the volume of gas entering previously poorly aerated and non-aerated lung regions after PEEP application	ZEEP vs PEEP 15 cmH_2_O	mL of recruited gas	Gas-based definition, conceptually distinct from tissue-mass–based recruitment
Chiumello et al., *Crit Care Med*, 2020 [62]	CT, tissue- and gas-based at high pressure	Recruitment assessed both as the reduction in non-inflated tissue and as the increase in lung gas volume between baseline and recruitment maneuver	5 vs 45 cmH_2_O (CT during recruitment maneuver)	Δ non-inflated tissue; Δ gas volume	High-pressure protocol yields larger recruitment estimates, limiting comparability with low-PEEP studies

*NA* non-aerated, *PA* poorly aerated, *ZEEP* PEEP 0 cmH_2_O, *FRC* functional residual capacity

Nevertheless, studies also highlighted the so-called “recruitability paradox”: similar global indices (e.g., compliance) may reflect very different regional distributions of aeration, so reliance on global mechanics alone can potentially mislead PEEP selection and lead to injurious ventilation in some patients [63]. qCT overcomes this limitation by showing where potentially recruitable tissue is located within the lung [64]. Clinically, greater recruitability may be associated with improved aeration at higher PEEP or during recruitment maneuvers. When recruitability is limited, however, increasing airway pressure may mainly lead to overdistension rather than additional lung reopening.

CT imaging also enabled mapping of regional opening and closing pressures across the lung, showing that recruitability is not uniform but varies according to local parenchymal properties. These range from areas of more resistant (“sticky”) to more easily recruitable (“loose”) atelectasis, helping to clarify the physiological basis of graded PEEP responsiveness [64].

Furthermore, the recruitment-to-inflation (R/I) ratio was evaluated against CT as a bedside surrogate of recruitability. In clinical studies using low-dose CT as reference, the R/I ratio demonstrated only modest accuracy in identifying highly recruitable patients, with correlation weaker in focal ARDS where PEEP might preferentially induce overdistension rather than true recruitment [65]. However, experimental models of highly recruitable lungs have shown that the R/I ratio correlates closely with CT-measured reductions in dynamic strain, supporting its physiological validity under well-controlled conditions [66].

Beyond static estimates of recruitable lung mass, CT imaging has been used to characterize tidal changes in lung aeration and deformation across the respiratory cycle using paired inspiratory and expiratory acquisitions. Parametric response mapping (PRM) is based on deformable image registration, a computational process that aligns two CT scans of the same lung obtained at different lung volumes, typically end expiration (EE) and end inspiration (EI), so that corresponding anatomical regions are spatially matched despite large changes in lung shape and position with inflation [67]. This step is essential to distinguish true regional changes in aeration from simple voxel displacement.

After registration, PRM enables voxel-wise comparison of lung density expressed in HU between EE and EI. This approach allows classification of lung tissue into regions with relatively stable aeration, persistently non-aerated consolidation, and areas showing large cyclic density changes between EE and EI, termed unstable inflation. Image registration also provides regional deformation metrics, most notably the Jacobian determinant of the transformation field, which quantifies local volumetric expansion or contraction between respiratory phases (Jacobian > 1 indicating expansion and < 1 compression).

PRM was first validated in experimental models of lung injury, where regions exhibiting unstable inflation were shown to be associated with the subsequent spatial propagation of secondary lung injury during mechanical ventilation. Prone positioning reduced the accumulation of unstable inflation in dependent regions and limited injury progression across animal models [68]. Similar observations were reported in humans. A retrospective analysis of ARDS patients demonstrated that a higher baseline fraction of lung tissue exhibiting unstable inflation was associated with worse outcome: values > 28% were observed in all non-survivors, whereas all survivors had ≤ 28% unstable tissue [69]. More recent clinical studies using CT registration have extended these concepts by integrating density- and deformation-based metrics, reporting associations between these image-derived functional variables and mortality in mechanically ventilated COVID-ARDS patients [70].

**Fig. 4:**
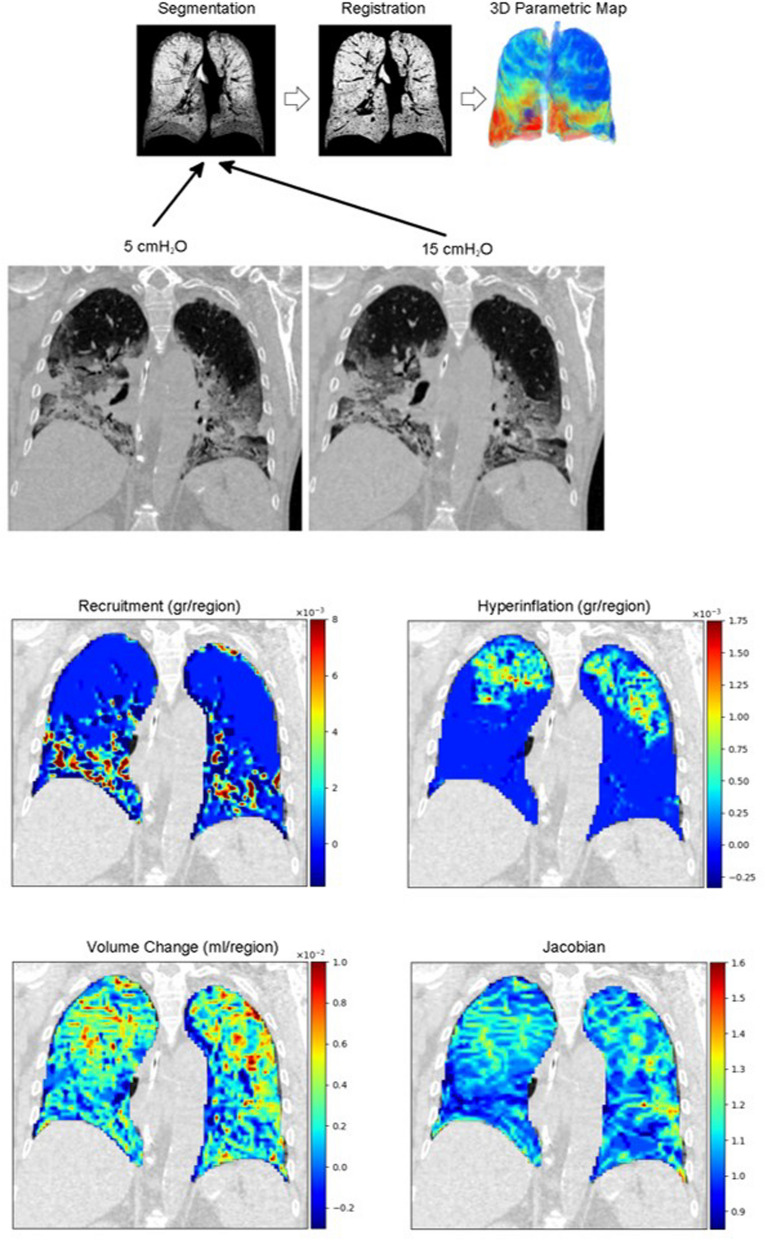
Lung functional imaging based on elastic registration of CT images acquired at static pressures of 5 and 15 cmH_2_O. A Image processing workflow. Inspiratory and expiratory CT images are segmented, then one image is warped using elastic image registration to perfectly match the other image. The indices of regional lung function are computed based on the registered images. B Sample parametric maps in an ARDS patient; recruitment, hyperinflation, and volume change are expressed per 4 × 4 × 4 voxel regions. Jacobian: determinant of the Jacobian matrix of the local deformation: Jacobian > 1: expansion, Jacobian < 1: contraction. Figure based on [70]

Recently, machine learning models were trained on CT-defined recruitability, measured as the change in non-aerated tissue between 5 and 45 cmH_2_O. The resulting model was then used to predict recruitability from a single CT acquired at 5 cmH_2_O. The promising accuracy of this approach suggests that lung recruitability may be inferred from a single low-pressure CT, potentially avoiding the need for repeated CT scans to quantify recruitment, with lower cumulative radiation exposure, fewer protocol-dependent sources of variability, and broader applicability compared with multi-pressure CT-based approaches [71] (Fig. 4).

Beyond quantitative segmentation, structured visual assessment of CT scans by experienced readers showed close agreement with quantitative methods and accurately classified high versus low recruitability, supporting pragmatic, standardized CT subphenotyping in routine clinical practice without the need for advanced software pipelines [72].

### Cardiopulmonary resuscitation-associated lung edema (CRALE)

**Fig. 5:**
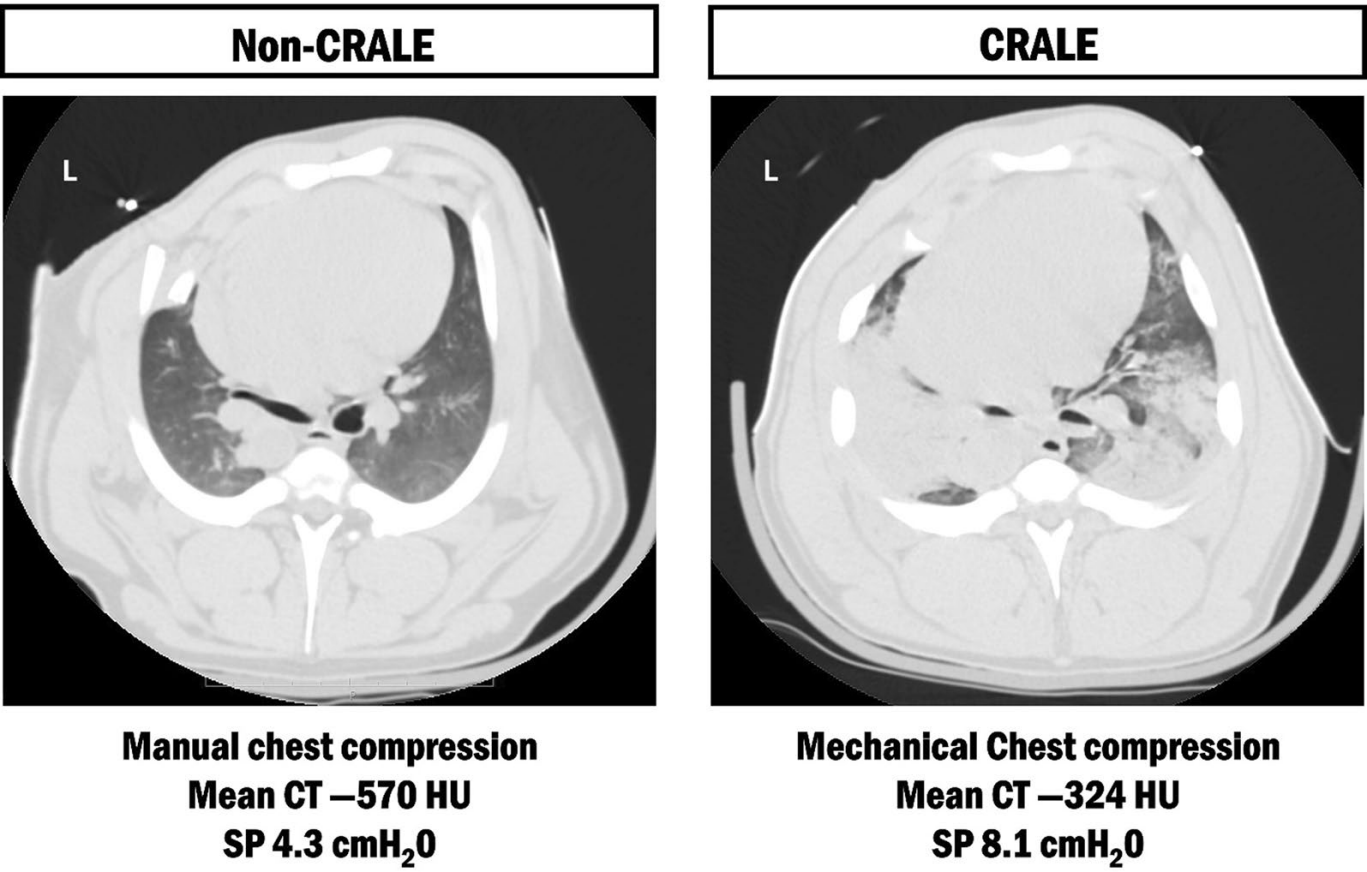
Representative axial chest CT images after cardiopulmonary resuscitation with different compression modalities. Left: manual chest compressions, without evidence of cardiopulmonary resuscitation-associated lung edema (CRALE), with lower mean lung density and preserved aeration. Right: mechanical chest compressions, with CRALE, characterized by higher mean lung density (higher mean HU values), increased non-aerated lung tissue, and higher superimposed pressure (SP)

In addition to ARDS-focused CT phenotypes, computed tomography has proven particularly informative in the identification and characterization of lung injury following cardiac arrest and resuscitation, a condition referred to as cardiopulmonary resuscitation-associated lung edema (CRALE). In a translational study that combined an animal model with an out-of-hospital patient cohort, CRALE was identified as pulmonary damage associated with chest compressions. Notably, mechanical chest compressions were linked to more severe injury than manual chest compressions as evidenced by increased lung weight, reduced compliance and oxygenation, in the presence of a higher intrathoracic pressure swing, and a higher incidence of abnormal lung density in patients, particularly those exposed to longer resuscitation [73] (Fig. 5). Quantitative CT features of CRALE include increased estimated lung weight, a higher proportion of non-aerated tissue, and a gravity-dependent distribution of abnormalities, which together are consistent with edema rather than simple atelectasis [74]. This was even supported by a rapid normalization of oxygenation within 3 h after return of spontaneous circulation. Furthermore, CT-defined CRALE relates to airway closure physiology [75, 76]: in a porcine arrest model, the airway opening index (AOI) captured differences in airway patency during mechanical versus manual compressions and was linked to lung structural changes, while clinical and experimental work has shown that intrathoracic airway closure during chest compressions limits delivered ventilation and shapes the CO_2_ signal [77, 78]. Importantly, the CT pattern is not merely descriptive. Patients meeting CRALE criteria showed lower respiratory system compliance and higher dead space than those without CRALE, with no relevant differences in chest wall mechanics [79].

The recently published 2025 guidelines from the European Resuscitation Council and European Society of Intensive Care Medicine [80,81] emphasize the importance of incorporating whole-body CT scan as part of post-ROSC care. Specifically, in comatose patients following cardiac arrest without ST-segment elevation and without a clear cardiac cause, an immediate whole-body CT scan is now recommended to identify the underlying cause and potential complications of cardiopulmonary resuscitation. This approach is particularly relevant as it may enhance the detection of CRALE and support more individualized ventilatory management in the post-ROSC phase based on CT-derived findings.

## Biological subphenotypes identified by latent class analysis (LCA)

LCA was introduced in ARDS to explore heterogeneity within the syndrome, acknowledging that patients meeting the same clinical criteria may differ substantially in underlying biology, clinical course, and response to supportive therapies. This approach uses routinely available clinical variables and plasma biomarkers to identify unobserved (“latent”) patient groups defined by systematically different combinations of biological and physiological features.

Across multiple randomized ARDS datasets, LCA repeatedly identified two subphenotypes: hyperinflammatory and hypoinflammatory [82, 83].

Starting from these biological and clinical models, LCA has subsequently integrated chest CT variables. In invasively ventilated COVID-19 ARDS, LCA combining qCT at a standardized PEEP with respiratory mechanics and gas exchange identified a recruitable class and a non-recruitable class. The recruitable class had lower PaO_2_:FiO_2_, lower compliance, less normally aerated lung, more non-aerated mass, and higher mechanical power at baseline; after a standardized recruitment maneuver, the more recruitable phenotype showed a larger decrease in non-aerated mass but longer time on the ventilator before successful extubation. While survival did not differ between classes, the sample size was probably underpowered to detect mortality differences [84].

Another analysis integrating CT-derived gas and tissue volumes with mechanics and gas-exchange variables also identified two latent classes. The non-recruitable class had lower respiratory system elastance (or higher respiratory system compliance), lower alveolar dead space, and less potentially recruitable lung than the recruitable class. With a standardized recruitment maneuver, the recruitable class increased ventilated lung tissue, compliance, and PaO_2_:FiO_2_ and reduced alveolar dead space; the recruitable class also had higher intensive care unit (ICU) mortality in adjusted analyses [85].

A multicenter veno-venous ECMO (vv-ECMO) cohort used CT and clinical variables at ECMO initiation and identified three subphenotypes labeled dry, wet, and fibrotic. 90-day in-hospital mortality was highest in the fibrotic group. A PEEP of at least 10 cmH_2_O during the first 3 days of ECMO was associated with lower mortality only in the wet group, with a significant PEEP interaction [86].

**Fig. 6:**
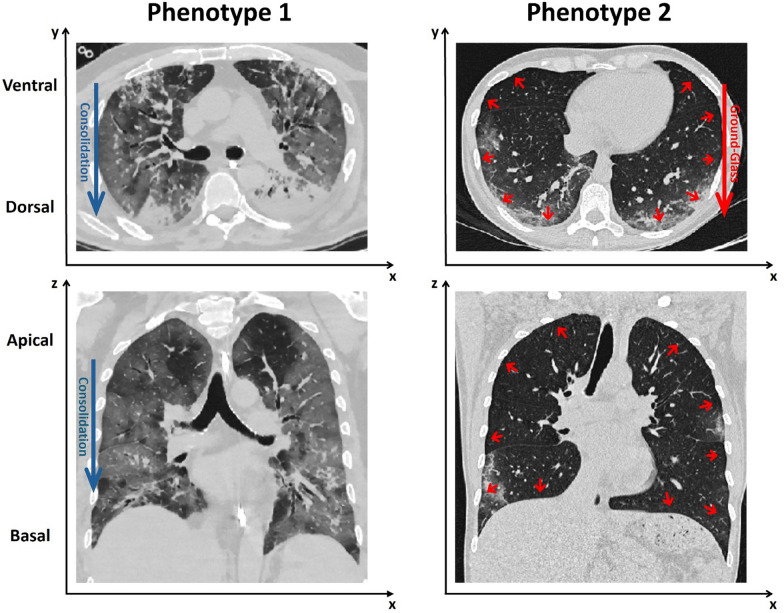
Representative axial (upper panels) and coronal (lower panels) chest CT images from spontaneously breathing patients with COVID-19 respiratory failure illustrating two distinct imaging patterns identified by latent class analysis integrating deep-learning-derived CT features. Left panels: predominant gravity-dependent consolidations, mainly in dorsal and basal regions, resulting in steeper ventro–dorsal and apical–basal density gradients. Right panels: diffuse ground-glass opacities with a predominant submantellar-to-hilar gradient, characterized by a higher extent of ground-glass opacities in subpleural (submantellar) regions progressively decreasing toward the hilar areas, with relative sparing of dense consolidations. These patterns reflect imaging-defined subphenotypes across the spectrum of early acute respiratory failure rather than established, mechanically ventilated ARDS. Images adapted from [21]

More recently, in spontaneously breathing COVID-19 patients with respiratory failure evaluated early after admission, LCA integrated deep-learning CT features with clinical and laboratory data and identified two subphenotypes. Importantly, patients were not invasively ventilated at the time of imaging and did not necessarily fulfill established ARDS criteria. Compared with the other class, one subphenotype was older, more hypoxemic, and had higher inflammatory biomarkers while its lungs showed a steeper gravitational density gradient with more consolidation and higher 90-day mortality. The other subphenotype showed a stronger submantellar-to-hilar density gradient with a greater proportion of ground-glass opacities. Differences were localized along three three-dimensional spatial trajectories: apicobasal, ventrodorsal, and centroperipheral (Fig. 6) [21]. These phenotypes were derived in spontaneously breathing patients early in the course of COVID-19 respiratory failure and therefore represent imaging-defined patterns across the acute respiratory failure spectrum rather than established ARDS phenotypes.

In addition to clustering approaches, emerging evidence indicates that specific qCT metrics provide independent prognostic information and thereby enhance models based solely on clinical and laboratory variables. In a large multicenter cohort of spontaneously breathing patients with COVID-19 acute respiratory failure, the inclusion of early CT-derived measures of lung involvement significantly improved mortality prediction. Among the CT descriptors examined—including laterality of infiltrates, the number of affected quadrants, and the global gas-to-tissue ratio—estimated superimposed pressure (SP) proved to be the most informative. SP quantifies the gravitational load imposed by increased lung density; as such, it helps integrate the contribution of lung density and lung height to injury severity within the underlying anatomical configuration of the lungs. SP emerged as the CT-derived parameter that best distinguished two distinct subphenotypes of lung injury and provided the strongest model fit for predicting 90-day mortality. This study represented the first evidence linking SP to clinical outcomes in non-intubated patients.

Taken together, these findings suggest that qCT can meaningfully contribute to risk stratification not only through complex multivariable techniques such as LCA, but also through single, physiologically grounded metrics which capture fundamental features of lung injury severity [87].

## Dual-energy CT (DECT) and estimates of regional ventilation/perfusion match

**Fig. 7:**
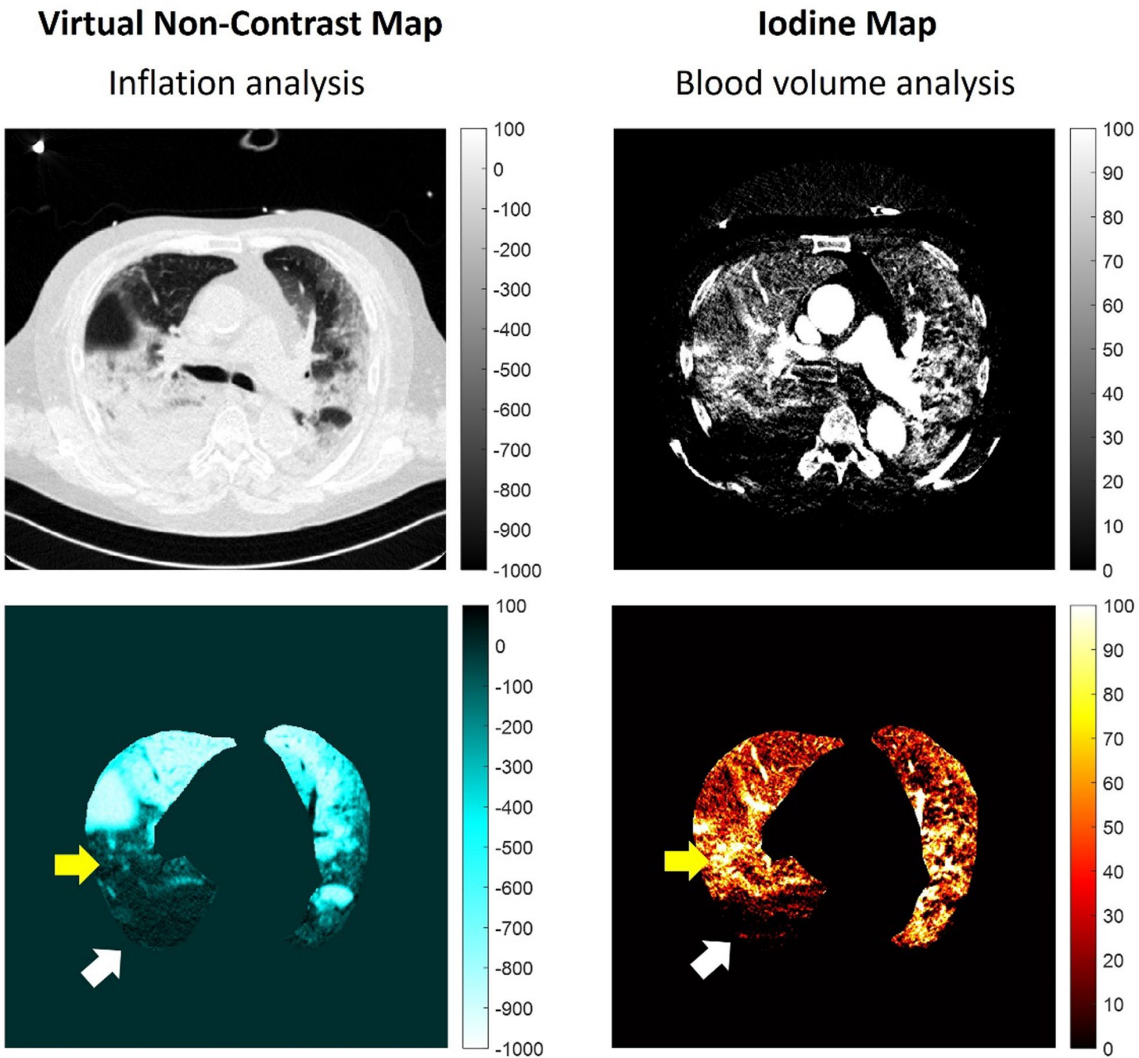
DECT COVID-19 ARDS patient. A representative example of a reconstructed image at the carina level, part of a static chest DECT scan. Left: virtual non-contrast (VNC), no-contrast agent CT reconstruction, used for inflation/collapse analysis (as a surrogate index of ventilation). Below: colormap highlighting non-inflated regions (-100 to +100 HU) in black and hyperinflated regions (-900 to -1000 HU) in white. Right: iodine map reconstruction, used for blood distribution analysis (as a surrogate index of perfusion). Below: colourmap highlighting perfused regions (0 to +100 HU) in black and not perfused areas (lower or equal to 0 HU). In this specific case, there are both inflation and perfusion defects, mainly represented in the dorsal region of the left lung (white arrows). At the same time, a hyperperfused region corresponds to a hypo/not inflated lung region in the middle portion of the left lung (yellow arrows). Clinical data: 90kg, male, 68 y. NIV (PSV) P_insp_ 2 cmH_2_O, PEEP 6 cmH_2_O. Vt/PBW = 8.9 mL/kg, RR = 19/min. ICU stay 3 days. Discharged alive.

DECT acquires images using two X-ray energies during a single contrast-enhanced study [88, 89]. Because iodine exhibits energy-dependent attenuation, the dual-energy information allows material decomposition and separation of iodine from background lung tissue. From this process, iodine distribution maps are generated, which reflect regional perfused blood volume (PBV) and serve as a surrogate of regional perfusion [90]. Areas with reduced iodine signal indicate impaired perfusion, whereas preserved signal suggests maintained blood flow. Importantly, the same acquisition can be post-processed to generate a virtual non-contrast image by mathematically subtracting the iodine component. This enables simultaneous assessment of lung aeration and perfusion within a single examination, without the need for separate non-contrast and contrast scans [91] (Fig. 7).

Traditional CT assesses the parenchyma well but does not directly measure regional pulmonary blood flow. In many patients with ARDS, ventilation–perfusion mismatch plays an important role in gas exchange impairment. DECT provides simultaneously acquired iodine-based maps of regional PBV and the corresponding virtual unenhanced images, the latter corresponding to aeration maps, thereby allowing identification of subphenotypes of ventilation and perfusion surrogates which are not evident on aeration maps alone [92].

Using this approach, DECT perfusion imaging demonstrated perfusion abnormalities in COVID-19 pneumonia, findings that are consistent with pathological evidence of endothelial injury, microthrombosis, and angiogenesis [93, 94]. Quantitative DECT studies in critically ill COVID-19 patients documented widespread perfusion abnormalities that often did not colocalize with aeration loss [92, 95]. In analyses comparing regional gas and blood-volume distributions, many patients showed perfusion defects within apparently normally aerated or ground-glass regions, consistent with microvascular obstruction or dysregulated pulmonary vascular tone; global PBV abnormality indices correlated with the severity of hypoxemia and the need for invasive ventilation [92]. These in vivo observations aligned with pathophysiological and post-mortem reports of endothelial injury, microthrombosis, and aberrant angiogenesis [96]. DECT cohort and retrospective studies extended these findings by directly comparing healthy controls, classical ARDS, and COVID-19; they identified distinct PBV signatures in COVID-19, with defects persisting beyond the acute phase and associated with elevated D-dimer and endothelial/inflammatory markers [92, 97, 98].

Finally, higher-intensity anticoagulation and, selectively, thrombolysis altered PBV maps and, in some patients, improved oxygenation [92, 99]. Corticosteroids were likewise associated with PBV redistribution and lower CT-derived lung weight on DECT [95]. Prospective studies remain necessary to confirm causality and potential outcome benefit.

In parallel, a multicenter physiological study of invasively ventilated COVID-19 ARDS obtained pulmonary CT angiograms in a subset of patients and reported complementary evidence of vascular involvement. Patients with markedly elevated D-dimer concentrations had higher ventilatory ratios (VR), defined as: $VR=\frac{\text{Minute Ventilation} (mL/min)*Pa{CO}_{2}(mmHg)}{\text{Predicted Body Weight}*100 (mL/min)*37.5 (mmHg)}$ [100], which is a surrogate of increased dead space. Furthermore, pulmonary CT angiograms frequently showed bilateral, diffuse hypoperfusion. The combination of high D-dimers and low static compliance identified a subgroup with increased 28-day mortality, linking elevated dead space and reduced compliance to worse outcomes. Although CT imaging was available only in a subset, the presence of diffusely distributed hypoperfusion in patients with high D-dimer concentrations is consistent with observations from DECT studies reporting perfusion defects in COVID-19 [101]. In a retrospective analysis of 118 patients with COVID-19 ARDS, Bjarnadottir et al. [102] used DECT to show that higher body mass index was associated with a better ventilation–perfusion match, consistent with the previously described “obesity paradox”.

Beyond COVID-19, experimental studies using DECT have shown that different inflammatory ARDS subphenotypes exhibit distinct responses to supportive treatments, including prone positioning and varying PEEP levels [103].

## Role of AI in lung CT imaging

Quantitative analysis of lung CT requires reliable lung segmentation. Delineating lungs and lobes is a prerequisite to computations that relate to compartmental aeration, estimated lung weight, total gas volume, lesion burden, and airway and vascular metrics. Without accurate segmentations, downstream measurements are biased and difficult to reproduce [104]. Manual segmentation is reliable but time-consuming and can be subjective, especially when performed across multiple pressure levels or across disease progression. Substantial effort has been directed toward developing AI methods for automated lung segmentation [105, 106]. Segmenting injured lungs remains particularly challenging as consolidations and pleural effusions make the lung boundary less distinguishable from adjacent structures, and automated algorithms may therefore mislabel tissue. For this reason, manual refinements are often required after the initial automated pass [107, 108].

A multi-resolution convolutional neural network (CNN) was proposed to achieve accurate lung segmentation in the presence of lung injury. The approach involved training a low-resolution model to learn global lung shape features, followed by training a high-resolution model to refine the precise lung boundaries, significantly improving performance compared to single resolution models. The models were first pretrained on human chest CT and then fine-tuned on animal models of acute lung injury. In tests across different species and CT scanners, it accurately segmented injured lungs, enabling automated segmentation even with a limited number of training datasets [109].

Polymorphic learning was proposed to train CNNs on diverse datasets, including humans with fibrosis and multiple animal species with lung injury. The model demonstrated the ability to generalize to lobar segmentation in COVID-19 patients without requiring any labeled training data. This approach could be extended to achieve lobar segmentation in acute lung injury across both human and experimental animal models. Currently, no automatic tools exist for non-human lobar segmentation [110].

Another study used an unsupervised approach, meaning it learned without expert-drawn lesion boundaries [111]. The model evaluated many CT scans of diseased lungs and many scans of healthy lungs and learned the visual differences between the two groups. It then created a “healthy-looking” version of each diseased scan and compared it with the original, highlighting the abnormal areas. From these differences, the authors obtained a lesion map and quantified the extent of lung involvement, without requiring manual labels. This enabled consistent estimation of the extent of lung involvement on COVID-19 CT.

In addition to segmentation, prediction is increasingly being explored as an application of artificial intelligence in this field. In a multicenter ICU study, the authors built a two-step system: the CT was first processed with an automated segmentation model to outline and measure typical abnormalities (e.g., ground-glass, consolidation, fibrosis, pleural effusion), and those quantitative CT features were then combined with routine clinical data to estimate a patient’s early ARDS risk. The segmentation-first approach performed better than another model that attempted to predict risk directly from raw, unsegmented CT images, supporting the value of converting images into structured, lesion-level measurements before prediction. The framework was evaluated at the development site, at independent hospitals, and in a prospective cohort, demonstrating promising generalizability [112].

Other predictive applications of CT-based AI often based on quantitative CT and radiomics features have been explored in several settings. In trauma cohorts, radiomics extracted from the admission CT was used to predict subsequent ARDS development, suggesting that quantitative descriptors may capture early parenchymal patterns not evident on routine clinical assessment [113]. In sepsis cohorts, CT-based deep learning and radiomics frameworks were proposed for early prediction of ARDS and severity stratification [114]. In COVID-19 qCT metrics and radiomics-enhanced models were used to predict clinical deterioration and escalation to advanced support, including vv-ECMO [115]. Across these studies, predictive performance depends on cohort characteristics and validation strategy, and current evidence primarily supports risk stratification rather than treatment selection.

## Emerging imaging techniques: dynamic CT and four-dimensional CT (4DCT)

Dynamic CT has recently been explored as a means by which to move beyond static imaging and to characterize how the injured lung behaves throughout the respiratory cycle. Dynamic CT refers to any technique that acquires repeated images over time to capture temporal changes in physiological processes. This allows the direct visualization of physiological phenomena—such as tidal recruitment, regional strain, and collapse that occurs during expiration—that cannot be assessed with static CT.

**Fig. 8:**
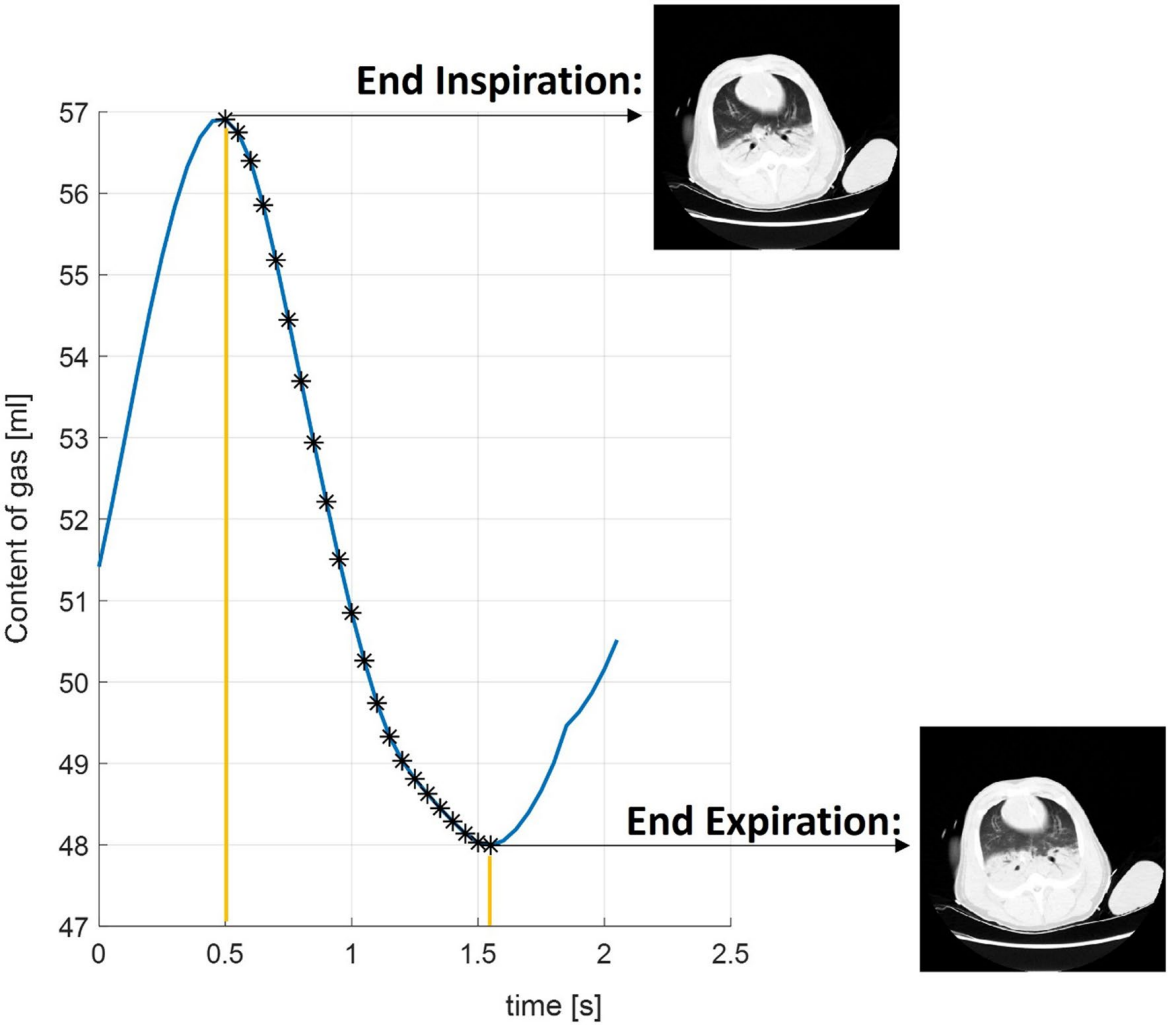
Dynamic CT. Representative example of volume changes (x-axis, content of gas reported in mL) from a 1-cm-thick image acquired dynamically at 20 Hz. Each asterisk (*) corresponds to one image detected during the expiratory phase

Depending on the specific CT modality used (e.g., conventional CT without contrast, Xenon-CT (Xe-CT), DECT, or PCCT), different physiological events can be investigated, including regional ventilation, ventilation–perfusion matching, and the passage of a contrast agent bolus. These techniques differ substantially in their achievable sampling rates, ranging from approximately 20 Hz (0.05 s per scan) with traditional CT (Fig. 8) to around 3 Hz with PCCT.

In the literature, the term “4DCT” is often used to describe dynamic CT acquisitions in which data are continuously acquired over time and subsequently reconstructed into multiple time points or respiratory phases using a respiratory signal, typically through respiratory-gated or phase-binned reconstructions. Importantly, the term 4DCT refers to the temporal dimension of image reconstruction rather than to a specific acquisition speed, and achievable temporal resolution varies widely depending on scanner technology, acquisition protocol, and z-axis coverage.

Most of the available evidence comes from animal models of ARDS. These studies showed that dependent regions often undergo marked intra-tidal changes in aeration and that regional deformation patterns vary substantially according to ventilatory mode and disease severity [116]. Other experimental work showed that poorly aerated tissue tends to deflate very quickly, supporting the concept that cyclic collapse in dependent regions contributes importantly to atelectrauma [117]. In murine models of ARDS, 4DCT was able to detect progressive increases in airway volume and anatomic dead space over only a few hours of mechanical ventilation, even in the absence of inflammatory injury [118]. In a swine model of moderate-to-severe ARDS, dynamic CT enabled the assessment of tidal airway closure and onset of absorption atelectasis during controlled ventilation [119]. A 20-Hz dynamic acquisition allowed detailed characterization of inspiratory transients of air redistribution [120] and their modulation by different inspiratory resistances and ventilatory settings. Dynamic CT further revealed the timing of atelectasis formation throughout expiration, demonstrating a diaphragmatic braking mechanism that limits expiratory flow and mitigates tidal opening and closure under conditions that promote lung collapse [121, 122].

Human data using this novel technique remain limited. Initial feasibility studies in sedated, mechanically ventilated patients indicated that respiratory-gated 4DCT is feasible and that dynamic changes in regional ventilation measured by 4DCT closely match those obtained with bedside EIT [123]. Additional evidence from dynamic imaging in the ICU showed that 4DCT can reliably assess intra-tidal volume changes and correlate them with delivered tidal volume [124]. Early clinical applications also suggested that 4DCT may reveal ventilation abnormalities that are not apparent on static CT, such as aerated lobes that do not participate in tidal ventilation [125].

Despite these promising observations, 4DCT currently has important limitations. Radiation exposure is substantially higher than with low-dose qCT, and image acquisition requires more time than a static scan. The technique also depends on complex registration and reconstruction pipelines that are not yet standardized across centers, which limits its broader clinical use. For these reasons, 4DCT remains primarily a research tool.

As acquisition protocols become more dose-efficient and analytic methods continue to improve, 4DCT may complement existing imaging approaches by providing information on dynamic processes, such as regional strain, tidal recruitment, and derecruitment, and heterogeneity of expiratory emptying that are central to ARDS pathophysiology but cannot be assessed with static imaging alone.

## Limitations and future directions

An important limitation of CT-driven subphenotyping is the limited availability of prospective trials in which imaging phenotypes are used to guide therapy. Most evidence to date comes from observational studies or post hoc analyses, which limits our ability to translate CT-defined phenotypes into treatment decisions that improve patient outcomes. The LIVE trial represents the main prospective attempt to individualize ventilatory strategies based on lung morphology, but it also highlighted the practical and methodological challenges of phenotype-guided interventions. These findings suggest that the key limitation lies not in the conceptual relevance of imaging phenotypes, but in the difficulty of implementing reliable and reproducible phenotyping approaches in routine ICU practice. Part of the recent literature on imaging-based phenotyping comes from COVID-19 studies. COVID-19 is a specific clinical context, and the imaging patterns described in this setting may not entirely reflect those observed in other ARDS etiologies. The methodological approaches developed during this period may still extend beyond COVID-19.

In addition, several technical limitations need to be considered. A first, pragmatic consideration concerns radiation exposure, because repeated CT scans may result in a relevant cumulative dose. In this setting, low-dose CT offers a possible approach to mitigate radiation exposure [118]. Low-dose chest CT has been specifically evaluated in ARDS and shown to preserve both visual assessment and quantitative measurements of lung aeration and density compartments, while substantially reducing dose, supporting its use for serial imaging when clinically justified [126].

Second, quantitative CT metrics remain sensitive to acquisition and reconstruction parameters, including section thickness, reconstruction kernel, tube current, and iterative reconstruction. These factors directly influence lung densitometry and shift the boundaries of aeration compartments, limiting cross-study comparability unless protocols and parameters are explicitly standardized and reported [127].

Third, although automated lung and lobe segmentation now performs well in many contexts and can be applied to injured lungs, performance degrades in the presence of extensive consolidation, lobar collapse, and pleural effusions. Segmentation errors may misclassify tissue and bias downstream metrics such as aeration fractions and estimated lung weight [128].

Another practical barrier to serial CT in ARDS is the time, logistics, and risk associated with transporting critically ill patients out of the ICU. The use of mobile or portable CT systems during the COVID-19 pandemic demonstrated that diagnostic chest CT can be performed at the bedside in selected ICU settings [129]. However, current systems are still limited by image quality, availability, and integration into routine workflows.

From a translational perspective, not all CT-derived metrics are at the same stage of clinical maturity. Quantitative measures based on standard lung segmentation and density thresholds, such as aeration compartment analysis and estimation of lung weight, are technically feasible with currently available software and can be performed in specialized centers, particularly when CT is already clinically indicated. By contrast, more complex methodologies including recruitability quantified across multiple PEEP levels, deformable image registration to assess regional opening and closing behavior, parametric response mapping, and machine learning based predictive modeling currently remain primarily research tools. These approaches require dedicated processing pipelines, strict standardization of acquisition protocols, and further prospective validation before routine clinical integration [128].

In parallel, computational approaches are being explored to limit the need for repeated full CT acquisitions. Recent proof-of-concept work has shown that three-dimensional lung representations can be generated by integrating prior CT scans with physiological variables and low-dimensional imaging inputs, such as chest radiographs. In experimental ARDS models, these synthesized images were able to reproduce changes in lung volume and aeration associated with different ventilatory conditions, with good agreement with reference CT [130]. Although these methods remain experimental, they suggest a potential complementary strategy to reduce the frequency of serial CT examinations, with possible implications for radiation exposure and transport-related risk.

Finally, PCCT is a recent technological development studied in comparison with conventional CT in terms of image quality, spatial resolution, radiation dose, and spectral imaging. Unlike conventional energy-integrating detectors, PCCT records individual photons and their energy, which reduces electronic noise and allows acquisition at higher spatial resolution for a given radiation dose. The availability of intrinsic spectral information enables material decomposition and the generation of iodine maps and virtual non-contrast images from a single acquisition, without the need for dual-source systems [131, 132]. Early thoracic applications have shown improved delineation of small airways and subsegmental vessels, as well as technically robust iodine mapping, with radiation dose profiles comparable to or lower than those of conventional CT [133–136]. Dedicated studies are required to define standardized acquisition protocols, to assess the stability and reproducibility of PCCT-derived quantitative metrics, and to determine whether the combined morphologic and perfusion information provided by PCCT translates into clinically meaningful imaging phenotypes [137].

More broadly, future prospective studies should evaluate the longitudinal behavior of imaging-defined phenotypes, interobserver reliability of both qualitative and quantitative classifications, and the feasibility and clinical impact of phenotype-guided therapeutic strategies across centers.

## Conclusion

CT-driven subphenotyping has improved the description of heterogeneity in ARDS, offering reproducible information on aeration, density, and perfusion.

Available data indicate that incorporating CT-derived variables into clinical models can refine prognostication. This signal has been observed both in invasively ventilated and in spontaneously breathing patients, where CT provides information that cannot be obtained from respiratory mechanics. In this context, metrics such as superimposed pressure offer an objective estimate of the gravitational load related to tissue density and edema and hold potential to approximate aspects of lung mechanical stress at a time when direct measurements are unavailable. Unlike qualitative pattern-based classifications, these measurements are derived from voxel-level density distributions and are less susceptible to misclassification. Such guidance cannot be reproduced with chest radiography. While further validation is required, current evidence indicates that quantitative CT could complement clinical assessment when a detailed assessment of the extent and regional pattern of parenchymal involvement is required.

## Data Availability

No datasets were generated or analyzed during the current study.
